# 慢性粒-单核细胞白血病外周血单核细胞亚群分析的单中心研究

**DOI:** 10.3760/cma.j.cn121090-20250126-00045

**Published:** 2025-10

**Authors:** 淋 王, 敏明 李, 姣姣 白, 程新 邓, 萍 吴, 成伟 罗, 沛龙 赖, 建宇 翁, 欣 杜

**Affiliations:** 南方医科大学附属广东省人民医院（广东省医学科学院）血液内科，广州 510083 Department of Hematology, Guangdong Provincial People's Hospital（Guangdong Academy of Medical Sciences）, Southern Medical University, Guangzhou 510083, China

**Keywords:** 慢性粒-单核细胞白血病, 单核细胞亚群, 辅助诊断, Chronic myelomonocytic leukemia, Monocyte subsets, Supporting criteria

## Abstract

**目的:**

评估外周血单核细胞亚群在慢性粒-单核细胞白血病（Chronic myelomonocytic leukemia，CMML）诊疗中的应用价值。

**方法:**

回顾性纳入2020年6月1日至2024年12月31日期间就诊于广东省人民医院初诊CMML患者51例（依据WHO 2022年诊断标准），为研究组；以23例伴有外周血单核细胞增多（绝对值≥0.5×10^9^/L，比值≥10％）的非CMML髓系肿瘤患者为对照组。所有患者均进行骨髓细胞形态学、活检、染色体、基因等检查，明确诊断；同时采用流式细胞术检测外周血单核细胞亚群比例，分析CD14^+^CD16^-^（MO1）、CD14^+^CD16^+^（MO2）及CD14^low^CD16^+^（MO3）各亚群比例，比较组间差异并通过受试者工作特征曲线（ROC曲线）评估诊断效能。

**结果:**

研究组51例CMML患者中，外周血单核细胞MO1亚群比例明显高于其他髓系肿瘤患者（*P*＝0.027），而MO2、MO3亚群差异均无统计学意义（均*P*>0.05）。进一步分析发现，CMML患者MO1亚群符合WHO诊断标准MO1亚群阈值（MO1％≥94％）者共43例（84.31％）；另8例未达到WHO标准。MO3亚群低于Hudson团队所给出的MO3亚群阈值（MO3％≤1.13％）的患者共46例（90.20％）；另5例高于此阈值。进一步分析发现，8例不符合WHO标准的患者中，7例处于炎症状态；5例不符合Hudson标准的患者均处于炎症状态。后续ROC曲线显示本队列中MO1亚群的临界值为97.55％［曲线下面积（AUC）＝0.661，*P*＝0.027］，符合WHO标准。

**结论:**

外周血单核细胞亚群分析，尤其是MO1亚群，可较好辅助CMML疾病诊断，但需要排除炎症状态。

## Abstract (en)

**Objective:**

To evaluate the clinical value of peripheral blood monocyte subset analysis in the diagnosis and treatment of chronic myelomonocytic leukemia（CMML）.

**Methods:**

We retrospectively enrolled 51 patients newly diagnosed with CMML at Guangdong Provincial People's Hospital between June 1, 2020, and December 31, 2024, according to the WHO 2022 diagnostic criteria. Twenty-three patients with other myeloid neoplasms（excluding CMML）and peripheral monocytosis（absolute count ≥0.5×10^9^/L and percentage ≥10％）were included as the control group. All patients underwent bone marrow aspiration for examinations including bone marrow smears, biopsies, cytogenetics, and gene mutation analysis to establish a definitive diagnosis. Concurrently, flow cytometry was used to determine the proportions of peripheral blood monocyte subsets: classical（MO1, CD14⁺CD16⁻）, intermediate（MO2, CD14⁺CD16⁺）, and non-classical（MO3, CD14^low^CD16⁺）. Differences between the groups were compared, and diagnostic efficacy was evaluated using receiver operating characteristic（ROC）curves.

**Results:**

Among the 51 CMML patients, the proportion of the peripheral blood MO1 subset was significantly higher than that in patients with other myeloid neoplasms（*P*＝0.027）, whereas there were no significant differences in the MO2 and MO3 subsets（all *P*>0.05）. Further analysis revealed that 43（84.31％）of the CMML patients met the WHO diagnostic threshold for the MO1 subset（≥94％）, while the remaining 8 patients did not; 46 patients（90.20％）had MO3 subset proportions below the threshold proposed by Hudson（≤1.13％）, while the remaining 5 patients were above this threshold. In-depth analysis showed that among the 8 patients who did not meet the WHO criteria, 7 were experiencing inflammation. Similarly, all 5 patients who did not meet the Hudson criteria were in an inflammatory state. Subsequent ROC curve analysis of this cohort identified a cut-off value for the MO1 subset of 97.55％［Area Under the Curve（AUC）＝0.661, *P*＝0.027］, which aligns with the WHO criteria.

**Conclusion:**

Peripheral blood monocyte subset analysis, particularly MO1 subset analysis, can effectively assist in CMML diagnosis, but exclusion of inflammatory conditions is required.

慢性粒-单核细胞白血病（Chronic myelomonocytic leukemia，CMML）是一种起源于造血干细胞的恶性克隆性髓系肿瘤，其特征性表现为持续性外周血单核细胞增多[1]–[2]，预后较差（中位生存期不超过3年）[3]–[5]，并极易转化为急性髓系白血病（AML）（3～5年内15％～30％的CMML患者转化为AML）[6]–[8]。

WHO 2016版造血与淋巴组织肿瘤分类提到“持续性外周血单核细胞增多≥1×10^9^/L，其中单核细胞占WBC≥10％”作为CMML的诊断标准之一[9]。2022年WHO第五版造血与淋巴组织肿瘤分类修订了CMML的诊断标准，CMML可基于WBC分为两个亚型：外周血WBC<13×10^9^/L定义为骨髓增生异常型（MD-CMML）；外周血WBC≥13×10^9^/L定义为骨髓增殖型（MP-CMML）[10]–[11]。该分类指出CMML的诊断需排除其他血液系统肿瘤，并表现为持续（>3个月）的外周血单核细胞增多（绝对值≥0.5×10^9^/L，比值≥10％），且骨髓和外周血中原始细胞比例<20％[8],[10]，此外，引入了流式细胞术分析单核细胞亚群辅助诊断，根据外周血中单核细胞表面CD14和CD16的表达分为三个亚群：CD14^+^CD16^-^（经典型，MO1）、CD14^+^CD16^+^（中间型，MO2）及CD14^low^CD16^+^（非经典型，MO3），其中MO1亚群比例≥94％被列为支持性CMML诊断标准[12]–[14]。2018年Hudson等[15]提出可根据外周血MO3亚群占比≤1.13％辅助诊断CMML。2019年Ong等[16]用CD33、CD86、CD64、HLA-DR和CCR2标记外周血单核细胞，以弥补CD16和CD14在体外的不稳定性，帮助识别不表达CD16和CD14的单核细胞。2020年Tarfi等[17]在其研究中引入了非经典单核细胞标志物slan，提出当slan^+^ MO3％低于1.7％时，可以作为CMML诊断的一个重要支持条件。

目前临床上应用最为广泛的是以CD14、CD16双标法分析外周血MO1亚群占比[13],[15]，对提高CMML诊断的准确性具有潜在价值。然而，外周血单核细胞增多可能有多种原因，例如反应性、克隆性[18]，而CMML患者的外周血单核细胞形态与其他反应性或克隆性单核细胞无明显差异[19]，这给鉴别诊断带来一定难度，同时炎症可以改变外周血单核细胞亚群比例[20]，这使得CMML的诊断具有一定的挑战性。关于外周血单核细胞各亚群占比在CMML诊断与鉴别诊断中的综合效能评估，尤其是该诊断阈值在真实世界队列中的符合情况以及潜在影响因素（如炎症、感染或肿瘤状态）的系统性研究，目前报道仍然有限。此外，Hudson等[15]提出MO3亚群≤1.13％可能具有诊断意义，但其在中国CMML人群中的适用性、诊断效能及与MO1≥94％标准的比较尚需验证。

为更全面地评估外周血单核细胞亚群分析在CMML诊疗中的应用价值，本研究比较CMML患者与伴有单核细胞增多的非CMML髓系肿瘤患者MO1、MO2及MO3亚群比例的差异；评估本组CMML患者符合WHO推荐MO1阈值（≥94％）及Hudson团队提出MO3阈值（≤1.13％）的情况；探讨不符合阈值患者的临床特征（重点关注炎症状态）；并通过受试者工作特征（Receiver operating characteristic, ROC）曲线分析确定本研究中区分CMML的最佳MO1及MO3临界值，并与现有阈值进行比较。

## 病例与方法

一、病例

本研究为单中心横断面诊断研究。连续纳入2020年6月1日至2024年12月31日期间于广东省人民医院血液科接受单核细胞亚群分析且资料完整的CMML患者。CMML的诊断与分型参照2022年第五版WHO造血与淋巴组织肿瘤分类。纳入本研究的患者还需排除以下情况：①有其他已知原因导致的单核细胞增多症，如感染、肿瘤（非血液肿瘤）所致的单核细胞增多症；②有严重的合并症或并发症，可能影响研究结果。另选取同期就诊、且外周血单核细胞增多的23例非CMML髓系肿瘤患者作为对照组：骨髓增生异常综合征（Myelodysplastic neoplasms，MDS）15例、骨髓增殖性肿瘤（Myeloproliferative neoplasms，MPN）3例、AML 5例。

二、外周血单核细胞亚群检测

留取患者外周血3 ml于EDTA抗凝管中，采集24 h内处理。加入1 ml 1×红细胞裂解液，室温避光裂解10 min，至溶液澄清，离心后弃上清；加入3 ml PBS洗涤细胞，离心后弃上清；用抗人CD14 APC-A、CD16 APC-Cy7-A荧光抗体标记单核细胞，室温避光孵育20～30 min；离心后弃上清；加入500 µl PBS重悬细胞，使用FACS Canto流式细胞仪（BD公司产品，美国）分析不同单核细胞亚群占比。亚群定义为：MO1（CD14^+^CD16^-^）、MO2（CD14^+^CD16^+^）及MO3（CD14^low^CD16^+^）。

三、二代测序

无菌采集CMML患者2～4 ml骨髓于EDTA抗凝管中，经Ficoll密度梯度离心分离单个核细胞，采用QIAamp DNA Blood Mini Kit提取基因组DNA。使用Covaris S220超声破碎DNA（目标片段150～200 bp），经NEBNext Ultra Ⅱ试剂盒进行末端修复、加A尾并连接Illumina UDI接头。采用IDT xGen Pan-Cancer Panel杂交捕获目标基因（含ASXL1、TET2、SRSF2等，65 °C 16 h），磁珠纯化后通过qPCR定量文库。在Illumina NovaSeq 6000平台进行PE150测序。生信分析包括：FastQC质控、BWA-MEM比对hg38参考基因组、GATK检测SNV/Indel、CNVkit分析拷贝数变异，经ANNOVAR注释后过滤（深度≥50×，VAF≥5％，排除gnomAD东亚人群频率>0.1％的多态性），最终通过Sanger验证致病突变（ACMG Class 4/5）。全程设置阴性对照。

四、统计学处理

连续型变量以“中位数（四分位距）”表示，分类变量以“例数（构成比）”表示。通过分析数据类型，确定CMML组和对照组不同单核细胞亚群之间的组间比较使用Mann-Whitney *U*检验。双侧*P*<0.05为差异有统计学意义。使用Graphpad Pism 9.5、SPSS Statistics 25.0进行数据分析及作图。绘制不同单核细胞亚群ROC曲线，并计算其诊断阈值及曲线下面积（AUC）。

## 结果

一、临床特征

本研究共纳入51例CMML患者，中位年龄66（范围：36～81）岁，男35例（68.6％）。其中36例（70.59％）外周血WBC<13（2.17～12.90）×10^9^/L，为MD-CMML。15例（29.42％）外周血WBC≥13（13.30～51.20）×10^9^/L，为MP-CMML。51例CMML患者中位单核细胞绝对值（Mono）为1.96（0.70～12.27）×10^9^/L，中位单核细胞比值（Mono％）为26.1％（10.3％～59.3％）。23例非CMML患者中位年龄65（15～90）岁，男20例（86.96％），中位Mono为1.16（0.50～69.72）×10^9^/L，中位Mono％为26.30％（11.10％～75.60％）。

二、CMML患者外周血单核细胞亚群

流式细胞术分析显示，相较于非CMML患者，CMML患者的MO1亚群比例显著升高［97.60％（*IQR*：2.90％）对96.10％（*IQR*：7.1％），*z*＝−2.208，*P*＝0.027］；而MO2、MO3亚群比例降低，且两组间差异无统计学意义［MO2：1.10％（*IQR*：1.50％）对1.50％（*IQR*：2.00％），*z*＝−1.069，*P*＝0.289；MO3：0.10％（*IQR*：0.30％）对0.20％（*IQR*：0.50％），*z*＝−0.119，*P*＝0.909］（图1）。

**图1:**
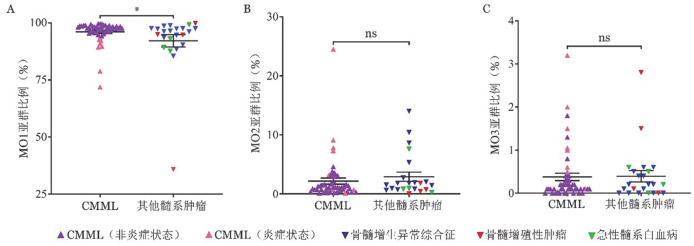
本队列CMML及其他髓系肿瘤患者单核细胞亚群分布情况（^＊^*P*>0.05，^ns^*P*>0.05） A MO1亚群；B MO2亚群；C MO3亚群 **注** CMML：慢性粒-单核细胞白血病

三、不同分类版本Mono标准对CMML诊断的差异

第四版CMML诊断标准中Mono≥1×10^9^/L，第五版CMML诊断标准中Mono≥0.5×10^9^/L，本研究51例患者中有10例外周血Mono≥0.5×10^9^/L但<1×10^9^/L，未达第四版标准。根据Mono是否满足第四版标准将患者分为两组，不同诊断标准下CMML患者MO1、MO2、MO3亚群比例差异均无统计学意义［MO1：97.60％（*IQR*：3.00％）对97.65％（*IQR*：2.97％），*z*＝−0.297，*P*＝0.775；MO2：1.10％（*IQR*：1.55％）对1.20％（*IQR*：2.63％），*z*＝−1.247，*P*＝0.218；MO3：0.10％（*IQR*：0.20％）对0.20％（*IQR*：0.84％），*z*＝−1.211，*P*＝0.231］（图2）。

**图2:**
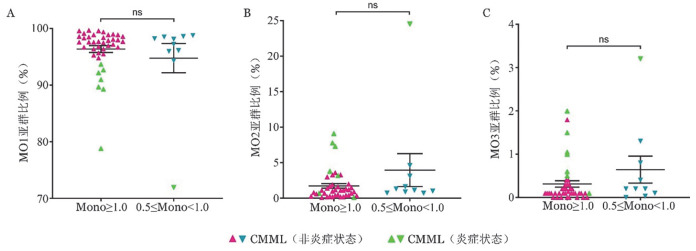
不同分类版本单核细胞（Mono）标准下CMML患者各单核细胞亚群分布情况（^ns^*P*>0.05） **A** MO1亚群；**B** MO2亚群；**C** MO3亚群 **注** CMML：慢性粒-单核细胞白血病；Mono≥1.0指单核细胞绝对值≥1.0×10^9^/L，即符合第四版CMML诊断标准；0.5≤Mono<1.0指单核细胞绝对值≥0.5×10^9^/L且<1.0×10^9^/L，即仅符合第五版CMML诊断标准但不符合第四版CMML诊断标准

四、CMML患者的基因突变情况

对所有CMML患者骨髓样本进行二代测序，重点分析表观遗传调控基因（ASXL1、TET2）及剪接因子基因（SRSF2）的突变特征。根据WHO标准将患者分为MO1％≥94％组（43例）及MO1％<94％组（8例）（表1）。比较两组基因突变频率，发现MO1％<94％组的患者ASXL1及TET2突变频率较高（62.50％，5/8）；MO1％≥94％组的患者，ASXL1突变占比较高（46.51％，20/43）。根据Hudson提出的MO3％界限值将患者分为MO3％≤1.13％组（46例）及MO3％>1.13％组（5例）（表1），MO3％≤1.13％组患者ASXL1及TET2突变占比较高，MO3％>1.13％组患者ASXL1突变占比较高。

**表1:** 不同分组标准的慢性粒-单核细胞白血病（CMML）患者与疾病高度相关的基因突变情况［例数（％）］

突变基因	WHO标准		Hudson标准	
	MO1％≥94％	MO1％<94％	MO3％≤1.13％	MO3％>1.13％
ASXL1	20（46.51）	5（62.50）	23（50.00）	2（40.00）
TET2	19（44.19）	5（62.50）	23（50.00）	1（20.00）
SRSF2	13（30.23）	4（50.00）	17（36.96）	0（0.00）

五、单核细胞亚群分析在CMML诊断中的应用分析

本研究51例CMML患者中符合第五版WHO补充标准MO1亚群阈值（MO1％≥94％）的患者共43例（84.31％），不符合WHO补充标准的患者8例，其中7例（87.50％）患者明确处于炎症状态（炎症指标CRP、PCT升高），另一例患者因临床资料缺乏，不能判断其临床状态；符合Hudson团队提出的MO3亚群减低（MO3％≤1.13％）阈值的患者共46例（90.20％），不符合MO3％≤1.13％阈值的5例（100.0％）患者均处于炎症状态。

六、不同单核细胞亚群的诊断效能对比

根据本研究纳入患者情况，绘制MO1、MO2及MO3亚群的ROC曲线并比较不同单核细胞亚群之间的诊断效能（图3）。ROC曲线分析证实MO1亚群的诊断价值（AUC＝0.661, *P*＝0.027），本队列MO1％最佳诊断阈值为97.55％，符合Selimoglu-Buet团队提出的≥94％标准；MO3％亚群阈值为0.015％，亦符合Hudson团队<1.13％标准。这表明国际诊断标准在中国人群中具有适用性。如图3所示，MO1亚群的AUC显著高于MO2与MO3亚群，诊断效能更为可靠。

**图3:**
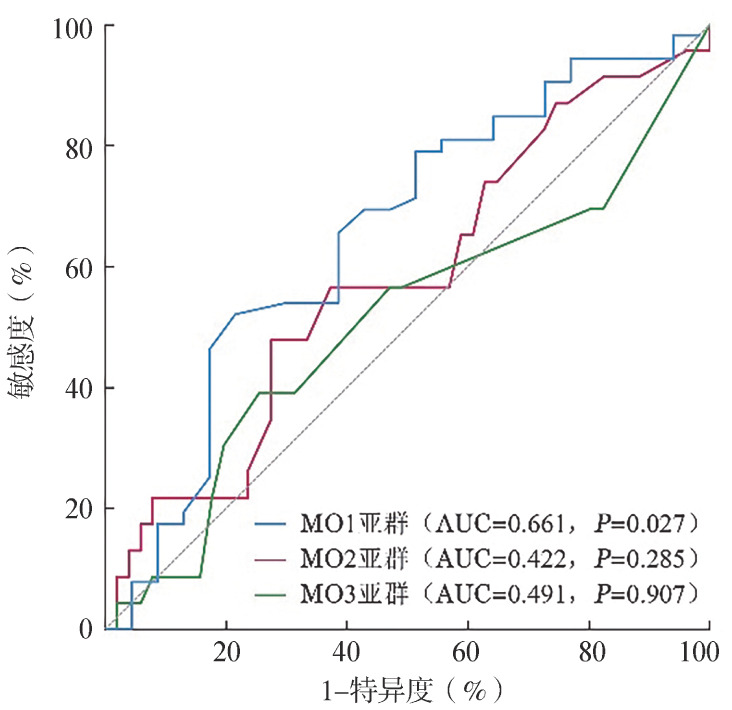
基于不同单核细胞亚群诊断慢性粒-单核细胞白血病（CMML）的受试者工作特征曲线 **注** AUC：曲线下面积

## 讨论

CMML是一种复杂的髓系肿瘤，其诊断依赖于多种检测方法的综合应用，同时需排除其他血液肿瘤及其他可导致外周血单核细胞慢性增多的感染、炎症、肿瘤等，诊断难度较大。近年来，随着Selimoglu-Buet团队提出应用流式细胞术分析外周血单核细胞亚群[12],[14]，辅助CMML诊断，越来越多的实验室开始研究CMML患者外周血单核细胞亚群特征。目前，应用最为广泛的是以外周血单核细胞中MO1亚群比例低于94％作为诊断阈值[13]，但目前对于其临床诊断效能的报道极少。

本研究纳入51例CMML患者，均有单核细胞绝对值及比例增加（中位Mono 1.96×10^9^/L，中位Mono％ 26.10％）。在临床应用中发现有非CMML的髓系肿瘤患者单核细胞也出现单核细胞增多。本研究对照组23例非CMML髓系肿瘤患者单核细胞绝对值及比例增加（中位Mono 1.16×10^9^/L，中位Mono％ 26.30％）。

本研究的CMML患者中，高频突变基因包括ASXL1、TET2、SRSF2，与既往国际研究报道的CMML核心突变谱一致。不同MO1亚群比例的患者突变基因分布略有差异：MO1高比例组（≥94％）以ASXL1突变为主，而低比例组（<94％）ASXL1及TET2的突变频率较高；MO3高比例组ASXL1突变频率较高，低比例组ASXL1及TET2突变频率较高。该结果提示，不同的基因突变类型或可影响单核细胞亚群的分布。

CMML患者队列中MO1亚群增多，其ROC曲线确定的最佳诊断临界值为97.55％，符合Selimoglu-Buet等提出的诊断阈值（MO1％≥94％），且展现出较高的诊断效能（AUC＝0.661）。相比之下，CMML患者MO2与MO3亚群比例减小，且MO2、MO3亚群在CMML组与非CMML组的差异无统计学意义（*P*>0.05）。进一步计算得出两组亚群的AUC均低于MO1亚群。本研究结果验证了Selimoglu-Buet团队提出的MO1诊断阈值（≥94％）在中国CMML人群中的适用性。

有研究指出当CMML患者处于炎症状态时，外周血单核细胞亚群分布可发生改变，MO1亚群可转化为MO3亚群[21]。我们在分析过程中也发现，有部分CMML患者的外周血MO1％未达WHO标准阈值，但MO3％增高。不符合MO1％标准的患者共8例，进一步分析这些患者的临床资料发现，87.50％（7/8）的患者处于炎症状态；不符合MO3％阈值的5例CMML患者也均处于炎症状态。因此，当患者处于炎症状态时，通过CD14和CD16分析外周血单核细胞亚群以辅助诊断CMML的准确性会显著降低，同时漏诊率明显升高。这一现象提示我们在检测前筛查炎症指标的重要性。炎症状态对CMML诊断具有干扰性[20]，排除炎症状态可以大大降低CMML患者的假阴性率。Selimoglu-Buet及Wagner-Ballon于2020年提出针对验证状态的诊断补充方案，当MO1％低于94％时，需要进一步分析MO3亚群，确定slan^+^的MO3亚群比例，slan^+^ MO3％低于1.7％可确诊为CMML[17]。

目前骨髓形态学在CMML的诊断中仍占据不可替代的地位，但其对实验技术人员的专业素质要求较高，主观因素影响较大，较难标准化，且流程复杂、耗时较长。流式细胞单核细胞亚群分析操作简单、快捷，可标准化，但因为其不能很好区分克隆性和反应性单核细胞的特征，目前仍不能取代骨髓形态学在CMML诊断中的主导地位。但在形态学难以鉴别时，流式细胞亚群分析可作为辅助手段，协助CMML诊断。

综上所述，与其他髓系肿瘤患者相比，CMML患者的MO1亚群比例明显增高，而MO2、MO3亚群比例则减低；相较于MO2、MO3亚群，MO1亚群标准的准确性更高；外周血单核细胞亚群分析可作为CMML疾病诊断的辅助诊断，尤以MO1亚群最为突出。炎症或可引起外周血单核细胞亚群之间的转化，因此，当患者存在炎症时，用MO1亚群辅助诊断CMML的准确性将大打折扣。

我们认为流式细胞术与骨髓形态学的联合应用能够显著提高CMML的诊断准确性，两者相互补充，能够为疾病的诊断提供更全面的信息。因此，在临床实践中，应根据患者的具体情况和诊断需求，灵活选择和组合这两种方法，以提高CMML的诊断水平。而我们需进一步探索更为精准的单核细胞免疫表型，以区分不同性质单核细胞，从而更好的服务于临床。
